# Characterization of Extracellular Vesicles from *Streptococcus thermophilus* 065 and Their Potential to Modulate the Immune Response

**DOI:** 10.1007/s12602-024-10422-0

**Published:** 2025-02-01

**Authors:** Angela Rocio Ortiz Camargo, Oscar van Mastrigt, Joost W. Gouw, Yue Liu, Roger S. Bongers, Jeroen van Bergenhenegouwen, Jan Knol, Tjakko Abee, Eddy J. Smid

**Affiliations:** 1 Food Microbiology, Wageningen University & Research, PO Box 17, 6700 AA Wageningen, The Netherlands; 2 Danone Research, Uppsalalaan 12, 3584 CT Utrecht, The Netherlands; 3 Laboratory of Microbiology, Wageningen University & Research, Wageningen, The Netherlands

**Keywords:** Lactic acid bacteria, *Streptococcus thermophilus*, Postbiotics, Proteomics, Phage, Immunomodulation, Cytokines

## Abstract

Bacteria can release membrane-derived nanoparticles made of lipid bilayers, so-called extracellular vesicles (EVs), which can carry diverse cargo and are important for microbe-microbe and microbe-host interactions. Here, we studied the production of EVs by *Streptococcus thermophilus* 065, the protein composition of the EVs, and how the produced EVs impact the immune response in vitro. Cultures of *S. thermophilus* grown for 6 h at 40 °C in M17 broth with 2% lactose reached high biomass yields and a high level of EVs quantified by lipophilic fluorescent dye staining. Proteome analysis of the isolated EVs revealed a high abundance of membrane-associated binding proteins of ABC transporters, ribosomal proteins, and glycolytic enzymes. In addition, phage proteins were found to be present in the EVs, which suggests a low-level expression of prophage genes during growth most likely supporting the release of EVs without causing cell lysis. The role of prophage activation was confirmed in an experiment with the addition of mitomycin C resulting in the expression of phage proteins including holin and endolysin causing a drop in culture OD and concomitant EV release. Subsequent in vitro immune assays using non-activated and activated human peripheral blood mononuclear cells (PBMCs) showed immune regulation in both cases upon exposure to *S. thermophilus* EVs and producer cells. This study shows the capacity of *S. thermophilus* EVs to act as immune modulators and opens the possibility for their use as postbiotics.

**Supplementary Information:**

The online version contains supplementary material available at 10.1007/s12602-024-10422-0.

## Introduction

Extracellular vesicles (EVs) are nanoparticles produced by cells in all domains of life including bacteria, and whose composition and biogenesis vary according to the producing organism [1].

The study of EVs in bacteria is gaining more attention due to the increasing awareness of the roles of the bacterial EVs in communication between microbes and their respective hosts including humans [2–6]. One of the presumed features of bacterial EVs is their capacity to activate an immune response in the host [7, 8], which includes the modulation of the production of anti and pro-inflammatory cytokines [9].

Both Gram-negative and Gram-positive bacteria can produce vesicles, and especially the studies on Gram-positive EVs accelerated in the recent decade [10–12]. The EVs produced by Gram-positive bacteria have a size distribution ranging from 20 to 300 nm and a wide diversity in cargo has been described including proteins, lipids, nucleic acids, and cell wall-derived exopolysaccharides and peptidoglycan components [10, 11].

Numerous studies have focused on the role of EVs produced by Gram-negative and Gram-positive bacteria including pathogenic *Streptococcus* [7, 8]. For example, EVs formed by *Streptococcus suis* contain subtilisin-like protease (SspA) and DNase that elicit an immune response by the host [13], and once internalized by endothelial cells cause pyroptosis by the presence of an inflammasome complex [14]. EVs from *Streptococcus pneumoniae* contain a wide range of proteins that can act as virulence factors including membrane-associated proteins, pore-forming proteins, metal ion and sugar transporters, and host-adhesion proteins [15, 16]*.*

*Streptococcus thermophilus* is one of the most commonly used beneficial bacteria in the dairy industry where selected strains are part of starter cultures for making fermented dairy products such as yogurts and different types of cheese [17] *S. thermophilus* is also used as a probiotic with reported health benefits associated to their consumption [18, 19]. In addition, health benefits of fermented formulas composed of combinations of *S. thermophilus* and *Bifidobacterium breve* have been reported and these benefits include alleviation of gut discomfort symptoms in infants and supporting the maturation of the immune system in new-borns [20–22].

In recent years, an increasing number of studies addressed the secretion and functionality of EVs produced by probiotic bacteria that showed diverse presumed health effects [23–25]. Nonetheless, there are no reports of the generation of EVs produced by *S. thermophilus* nor of their role in immune modulation. Thus, the aim of this study was to fill this knowledge gap and to identify and quantify the production of EVs in *S. thermophilus* 065, characterize their protein composition, and elucidate the possible role of EVs as immune modulators in in vitro studies using peripheral blood mononuclear cells (PBMCs).

## Materials and Methods

### Bacterial Strain

In this study, we used *S. thermophilus* 065, which was obtained from the Danone Culture Collection, Gif-sur-Yvette, France. Stock suspensions of this strain were stored at – 80 °C in M17 broth (BD Difco™) supplemented with 1% lactose (VWR Life Science, France) and with glycerol (30% (v/v)) until further use.

### Pre-culturing Conditions

A single colony of *S. thermophilus* 065 was inoculated in 10 mL M17 broth (BD Difco™) supplemented with 2% lactose (LM17; VWR Life Science, France). Overnight cultures of *S. thermophilus* were incubated at 42 °C for 24 h in anaerobic jars (Advanced Instruments, USA) treated with Anoxomat (Advanced Instruments, USA) to obtain a microaerophilic condition (max 6% O_2_). To obtain a higher number of bacteria for the subsequent batch cultures, the initial overnight cultures were propagated by transferring 5 mL to a second overnight culture with 50 mL of M17 supplemented with 2% lactose followed by incubation at the conditions mentioned above.

### Batch Cultures

Batch fermentations were carried out in 0.5 L bioreactors (Multifors, Infors HT, Switzerland) in biological triplicates. The bioreactors containing M17 broth (BD Difco™) supplemented with 2% lactose (LM17; VWR Life Science, France) were inoculated with an over-night culture (10% v/v) obtaining an initial optical density between 0.2 and 0.3. The initial pH was 6.5, the temperature was maintained constant at 40 °C and the stirring speed was 300 rpm. To maintain anaerobic conditions, the headspace was flushed with nitrogen gas at a rate of 0.06 L/min. To induce prophage expression after 1.5 h, mitomycin C from *Streptomyces caespitosus* (Sigma-Aldrich, USA) was added at a final concentration of 1 µg/mL. Samples were taken every 1.5 h for vesicle quantification and at the endpoint (after 6 h approximately) for proteomics, transmission, and scanning electron microscopy.

### Collection of Extracellular Vesicles by Ultra-centrifugation

For the proteomic and the immune analyses, samples of 300 mL were taken from the bioreactor and immediately centrifugated at 6000 × *g* for 15 min. The supernatants were then filtered through a 0.2-µm syringe filter (Minisart). Afterward, the samples were concentrated to 150 mL using Amicon® Ultra-15 centrifugal filter units of 100 kDa (Merk, Germany) by centrifugation at 5000 × *g* for 30 min. Then, the samples were ultra-centrifugated at 150,200 × *g* for 1 h at 4 °C in polycarbonate bottles (Beckman Coulter, USA) suitable for the ultracentrifuge Beckman Optima XE-90 (Beckman Coulter, USA). After centrifugation, the pellets were re-suspended in 600 µL of phosphate-buffered saline (PBS) buffer and stored at – 80 °C until further use.

### Collection of Extracellular Vesicles with Polyethylene Glycol (PEG8000)

For quantification purposes only, EVs were collected by the PEG precipitation method. Samples of 20 mL were taken from the bioreactor and centrifuged at 6000 × *g* for 15 min. The supernatants were then filtered with a 0.2-µm syringe filter (Minisart) to remove the remaining bacterial cells. To 20 mL filtered supernatant, 5 mL of precipitation buffer containing 20% PEG8000 (Sigma, Germany) and 2.5 M NaCl was added. This mixture was gently stirred and incubated overnight at 6 °C. EVs were recovered by centrifugation at 11,000 × *g* for 60 min at 4 °C. Afterward, the supernatants were discarded and the pellets were carefully drained from any remnant liquid. The pellets were gently resuspended in 0.2 mL of PBS and stored at – 20 °C until further use.

### Quantification of Extracellular Vesicles

To quantify the EVs, the membrane-selective fluorescent lipophilic dye FM4-64 (InvitrogenTM, USA) was used. Briefly, the dye FM4-64 was dissolved to a concentration of 10 µg/mL in PBS. Next, 50 µL of each precipitated sample was mixed with 50 µL of the dye suspension in a black polystyrene 96-well plate and incubated for 15 min at room temperature in the dark. Finally, samples were measured in a spectrophotometer at an excitation and emission wavelength of 515 and 640 nm, respectively. After removing the background signals from all samples, a relative comparison was made between samples measured in the same assay.

### Scanning Electron Microscopy (SEM)

The morphology and production of EVs from *S. thermophilus* 065 were investigated with SEM as previously described [26]. The bacterial samples were centrifuged at 17,000 × *g* for 1 min and the pellets were frozen at − 20 °C until use. On the day of analysis, samples were thawed and resuspended in peptone physiological salt (PPS). For each sample, a drop of the suspension was placed in a poly-L-lysine coated coverslip (Corning BioCoat, USA) and left for 1 h at room temperature. Then, the coverslips were rinsed with phosphate buffered saline, and fixed with 3% glutaraldehyde buffer for 1 h. Then, the samples were dehydrated in a graded series of ethanol followed by drying with CO_2_ (Leica EM CPD 300, Leica Microsystems, Germany). The coverslips were fitted onto sample stubs with carbon adhesive tabs and sputter coated with 10-nm tungsten (Leica SCD500). Lastly, samples were imaged at 2 kV, 6 pA, at room temperature in a field emission scanning electron microscope (Magellan 400, FEI Company, USA).

### Transmission Electron Microscopy (TEM)

The morphology of the EVs from *S. thermophilus* 065 was investigated with TEM as described by [12]. Briefly, EV samples were negatively stained prior to imaging. Thus, 2 µL of EV suspension was applied to a 400 mesh copper grid supplied with a formvar/carbon film and incubated for 2 min. Afterward, the grid was rinsed with 5 µL of Milli Q water. Then, the grid was stained with 2% uranyl acetate for 30 s and dried with filter paper. The samples were visualized with a Jeol JEM‐1400 plus TEM equipment (Jeol, Japan) with an accelerating voltage of 120 kV.

### Proteomic Analysis

For the proteomics analysis, samples of EVs and bacterial cells were taken from the bioreactor. The EVs were collected by ultra-centrifugation as mentioned above. Bacterial cells were collected by centrifugation for 15 min at 6000 × *g*. Cell pellets were washed three times with PBS by centrifugation for 5 min at 6000 × *g* and then cells were frozen with liquid nitrogen and stored at – 80 °C until further use. Before analysis, EV lysis was achieved by three cycles of sonication for 30 s in a Soniprep 150 ultrasonic disintegrator (MSE, UK) and 30 s of rest in ice in between. For the samples of bacterial cells, cell lysis was carried out with bead beating in a FastPrep-24 5G instrument (MP Biomedicals) in six cycles of 30 s at 6.5 m/s with cooling after every bead step. Protein quantification and analysis were carried out as described previously [26] Hence, protein quantification was initially carried out using the Pierce bicinchoninic acid (BCA) protein assay (Thermo Fisher Scientific, Waltham, MA, USA) to normalize the samples for both proteomic analysis and immune analysis. Protein digestion was performed overnight using dithiothreitol (DTT, 2 mM), iodoacetamide (IAA, 4 mM), and trypsin (1:50 of a 1 mg/mL solution) at 37 °C. Clean-up was performed with solid phase extraction (SPE) columns (WATERS) with formic acid (0.1% in water). Subsequently, samples were analysed by nano-liquid chromatography high-resolution mass spectrometry (nano-LC-HRMS/MS) as described in (Meiring et al. 2002). An UltiMate™ 3000 RSLCnano System (Thermo Fisher Scientific, Waltham, MA, USA) was used and connected to a Q-Exactive Plus mass spectrometer (Thermo Fisher Scientific). Peptides were trapped on a µ-Precolumn Cartridge (Acclaim Pepmap 100 C18, 5 µm, 100A 300 µm × 5 mm, Thermo Fisher Scientific) at 30 µL/min in 0.1% formic acid. Then, the peptides were eluted at 300 nL/min in a 90-min extended gradient from 7 to 40% formic acid solvent (in 80% acetonitrile) to a 15-cm bioZen 2.6 μm Peptide XB-C18, nano Column, 150 × 0.075 mm (00F-4782-AW-21, Phenomenex, Utrecht, The Netherlands). The acquired spectra were analysed using Thermo Proteome Discoverer (v3.1) in combination with Mascot (v2.5) (Thermo Fisher Scientific). The reference database comprised protein sequences from *S. thermophilus* 065 from UniProt and typical contaminants. Built-in percolator was used with default settings for postprocessing of Mascot peptide spectrum matches (PSMs) from Proteome Discoverer. In all experiments, PSMs were filtered to a peptide false-discovery rate of 1% using q values that were calculated based on PSM score distributions for decoy database searches as well as considering a minimum peptide length of six amino acids. Proteins were filtered to a protein false-discovery rate of 1% and a minimum requirement of two unique peptides. Relative protein quantification was performed with Proteome Discoverer based on peptide intensity signals using default settings.

### Proteome Data Analysis

To correct for differences in protein extraction efficiency, first, the minimum and sum of all protein quantities per sample were calculated (minimum protein abundance and total protein abundance, respectively). Subsequently, missing values were substituted by the minimum protein abundance and all protein abundances were normalized by dividing the protein abundances by the corresponding total protein abundance of that sample (normalized protein abundance). The normalized protein abundances were log10-transformed to make the variances more similar over the large range of abundance. To calculate fold changes, the average of the log10-tranformed abundances was calculated and subtracted. For the corresponding significance, paired *t*-tests were performed on the log10-transformed abundances. The abundance of proteins was considered significantly different with a p-value below 0.05 and a fold-change of more than 2 or less than 0.5. The normalized abundances of all detected proteins are listed in (Supplementary Table 1).

The detected proteins were assigned to different groups (glycolytic, ribosomal, phage, membrane, and other) based on the protein sequence information and their annotation. The group of glycolytic proteins were all the proteins involved in the metabolism of lactose to pyruvate, including the β-galactosidase and the enzymes for subsequent conversion of glucose and galactose to pyruvate. The group of ribosomal proteins consisted of exclusively predicted ribosomal proteins that are part of the small and large ribosomal subunits. The group of phage proteins contains all the proteins that were encoded by the two prophages (one complete and one satellite prophage). Finally, the group of membrane proteins consisted of all the proteins that were predicted to have transmembrane helices with TMHMM 2.0 [27, 28] and/or had predicted signal peptides by SignalP 6.0 [29] and were not part of the previously described groups. So, this group also includes proteins that were predicted to be secreted.

### Data Availability

The raw proteomic data supporting the conclusions of this article have been deposited on 4TU.ResearchData (https://data.4tu.nl/) with the digital object identifier: 10.4121/9d74337b-f44a-4d68-82db-c3b88662268d

### Immune Response Analysis

Peripheral blood mononuclear cells (PBMCs) were isolated from buffy coats obtained from healthy donors who had provided written informed consent (Sanquin, Nijmegen, The Netherlands). Buffy coats were diluted 1:1 with sterile phosphate-buffered saline (PBS) (Sigma-Aldrich) containing 2% heat-inactivated foetal bovine serum (FBS-Hi) (HyClone™ FBS, Fisher Scientific, Loughborough, UK) followed by density centrifugation using Greiner Bio-One™ LeucoSEP™ polypropylene tubes pre-filled with Leucosep separation medium. The interface layer, containing PBMCs, was isolated and the cells were washed three times in PBS containing 2% FBS. After the final wash, PBMCs were resuspended in RPMI 1640 with glutamine containing 2.5% FBS-Hi at a concentration of 2 × 10^6^ cells/mL. Ninety-six-well flat-bottom plates were seeded with 2 × 10^5^ cells/well and stimulated with *S. thermophilus* 065 intact bacteria at a concentration of 1 or 10 µg protein/mL or *S. thermophilus* 065 vesicles at 10 or 50 µg protein/mL or the microbial-associated molecular patterns (MAMP) PAM2 (Pam2CysSerLys4), PAM3 (Pam3CysSerLys4) (InvivoGen, UK) as microbial-product controls at 100 ng/mL. The amount of bacterial cells that correspond to 1 and 10 µg protein/mL samples is approximately 10^7^ and 10^8^ cells/mL, respectively. After 24 h incubation at 37 °C and 5% CO_2_, PBMCs were activated with 5 ng/mL CD3 (clone HIT3a, BD Biosciences, UK) and 5 ng/mL CD28 (clone CD28.2, BD Biosciences, UK) for another 24 h incubation after which cell-free supernatants were collected and stored at – 20 °C until analysis. Cytokine and chemokine content (CCL22, CXCL10, IL6, IL10, TNFα, CCL1, CCL17, CCL20, CXCL9, INFγ, IL4, IL13, IL17, MIF) in the cell-free supernatants was analysed using a Luminex assay (R&D Systems, USA) on a Luminex FLEXMAP 3D instrument system (Thermo Fisher, USA) according to the manufacturer’s instructions.

## Results

### Growth of *S. thermophilus* 065 and EVs Production

**Fig. 1:**
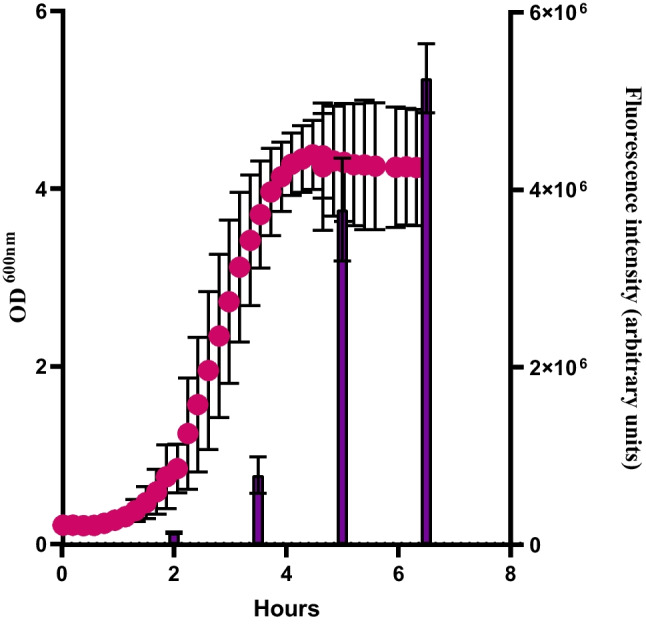
Growth curve of *S. thermophilus* 065 at 40 °C (red dots) and the corresponding EV production as indicated by the fluorescence of FM4-64 (purple bars). Error bars correspond to the standard error of the mean

To determine the production of EVs by *S. thermophilus* 065, this strain was grown in batch cultures at 40 °C in M17 broth supplemented with 2% (w/v) lactose while monitoring the optical density. Samples were taken throughout the fermentation process to quantify EVs using the fluorescent dye FM4-64, which is selective for membranes. The OD of the culture increased during the first 4 h of incubation and remained stable up to the final time point (Fig. 1). Samples taken at the indicated time points showed increasing fluorescence during growth reaching the highest level at the latest sampling time. This result suggests that EVs are continuously released during growth.

**Fig. 2:**
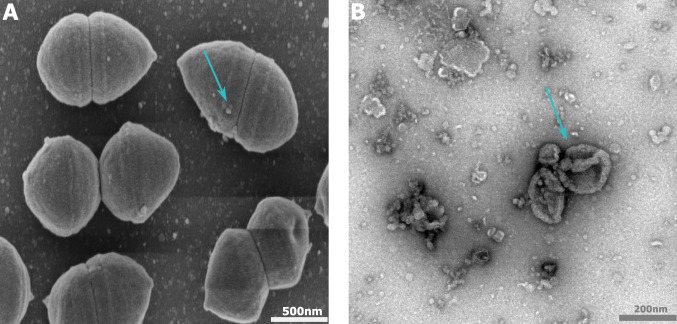
SEM image of *S. thermophilus* 065 cells with EV-like spherical structures attached (**A**), and TEM image of *S. thermophilus* EVs in suspension (**B**). Blue arrows point to EVs

To confirm that the increased fluorescence was indeed linked to the production of EVs, samples taken at the latest time point were analysed by scanning electron microscopy (SEM) and transmission electron microscopy (TEM) (Fig. 2). SEM images show intact *S. thermophilus* 065 cells with small spherical structures attached to the cells, conceivably representing EVs that are still attached to the cells (Fig. 2A). TEM analysis of EV preparations, obtained after filtration and ultracentrifugation as described in the methods section, shows vesicle-like structures with sizes in the range of 50–200 nm (Fig. 2B). In addition, some bacteriophage-like particles could be observed (Supplementary Fig. 1).

### Proteomic Analysis

**Fig. 3:**
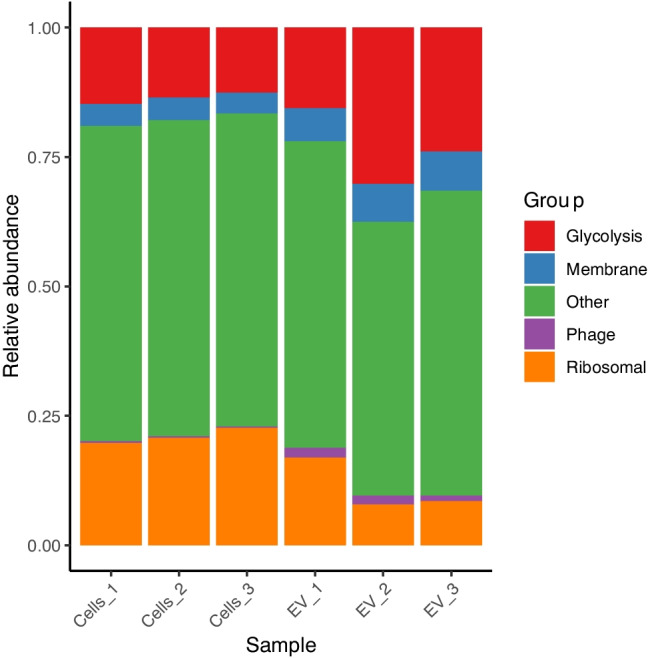
Relative abundances of the proteins in bacterial cells and EVs of *S. thermophilus* 065 obtained from cultures grown at 40 °C in M17 with 2% (w/v) lactose. Data are shown for three independent biological replicates

Comparative proteomics analysis of EVs isolated from *S. thermophilus* 065 and the corresponding cellular fractions were performed. In total 943 proteins were identified in the samples of *S. thermophilus* 065 or EVs, which were grouped as either glycolytic proteins, membrane proteins, phage proteins, ribosomal proteins, or others (Fig. 3, Supplementary Table 1). The EV fraction was quantitatively enriched in membrane-associated proteins and glycolytic proteins in comparison to the cellular fraction. In addition, bacteriophage-encoded proteins were found and enriched in the EV fraction.

**Fig. 4:**
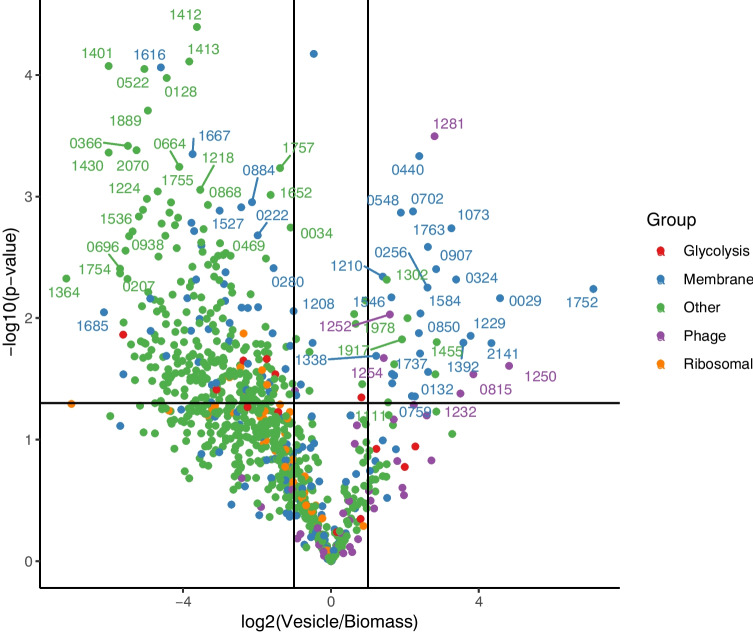
Volcano plot showing the differences in the abundance of proteins in the EVs fraction in comparison with the bacterial cells, with the top right quadrant presenting proteins enriched in EVs (see Table 1 for the description of all the EV-enrich proteins). Colours indicate to which group the protein belongs and numbers represent the last four digits of the accession number of the protein. A complete list of all detected proteins can be found in (Supplementary Table 1)

To further investigate which particular proteins were enriched in the EV fraction in comparison to the corresponding cells, differentially expressed proteins were visualized in a volcano plot (Fig. 4) and EV-enriched proteins are listed in Table 1.

**Table 1:** Enriched proteins in the EV fraction in comparison with the corresponding cells

Accession	Description	log2 (vesicle/biomass)	*p*-value
STHE65_v1_1752	Na + : H + antiporter	7.09	0.0058
STHE65_v1_1250	Phage head protein	4.82	0.0247
STHE65_v1_0029	Glucan-binding protein	4.57	0.0069
STHE65_v1_2141	Putative permease	4.34	0.0160
STHE65_v1_0815	DNA primase, phage associated	3.85	0.0289
STHE65_v1_1229	Protein of unknown function	3.78	0.0140
STHE65_v1_1392	Conserved exported protein of unknown function	3.58	0.0159
STHE65_v1_1232	Conserved protein of unknown function	3.50	0.0418
STHE65_v1_0324	Cysteine ABC transporter, substrate-binding protein	3.39	0.0048
STHE65_v1_1073	Iron compound ABC uptake transporter substrate-binding protein PiuA	3.26	0.0018
STHE65_v1_1455	Conserved protein of unknown function	2.86	0.0157
STHE65_v1_0907	Zinc-binding lipoprotein	2.84	0.0040
STHE65_v1_1308	3-Isopropylmalate dehydrogenase	2.82	0.0290
STHE65_v1_1281	Conserved exported protein of unknown function	2.80	0.0003
STHE65_v1_1737	O-sialoglycoprotein endopeptidase	2.63	0.0277
STHE65_v1_1763	Cysteine ABC transporter, substrate-binding protein	2.62	0.0026
STHE65_v1_0256	Penicillin-binding protein 1A	2.61	0.0056
STHE65_v1_1584	ABC transporter, substrate-binding protein	2.42	0.0092
STHE65_v1_0955	ABC transporter substrate-binding protein	2.41	0.0195
STHE65_v1_0440	Conserved protein of unknown function	2.39	0.0005
STHE65_v1_0850	Putative lipoprotein	2.37	0.0133
STHE65_v1_0132	D-alanyl-D-alanine-carboxypeptidase	2.27	0.0441
STHE65_v1_0702	Conserved protein of unknown function	2.22	0.0013
STHE65_v1_0759	Conserved exported protein of unknown function	2.19	0.0438
STHE65_v1_1978	Glutamyl aminopeptidase	2.07	0.0100
STHE65_v1_1917	Ribulose-5-phosphate 3-epimerase	1.92	0.0150
STHE65_v1_0548	Peptidyl-prolyl cis–trans isomerase ppiA	1.89	0.0014
STHE65_v1_1885	Signal peptidase I	1.71	0.0297
STHE65_v1_0230	Chaperonin large subunit groEL	1.71	0.0240
STHE65_v1_0145	Oligopeptide-binding protein AmiA	1.66	0.0344
STHE65_v1_1314	Conserved protein of unknown function	1.65	0.0287
STHE65_v1_1546	Oligopeptide-binding protein	1.64	0.0068
STHE65_v1_1252	Conserved protein of unknown function	1.60	0.0093
STHE65_v1_1111	Conserved protein of unknown function	1.55	0.0493
STHE65_v1_1302	6-Phosphofructokinase	1.51	0.0048
STHE65_v1_1254	Portal protein	1.43	0.0213
STHE65_v1_1210	DNA-entry nuclease	1.40	0.0045
STHE65_v1_1338	Conserved membrane protein of unknown function	1.22	0.0204

Notably, the proteins that were most enriched in the EVs included membrane-associated substrate-binding proteins of putative oligopeptide, cysteine, methionine and cobalt/B12 ABC-type transporters, enzymes involved in cell wall synthesis/turnover including penicillin-binding protein PonA, glucan-binding protein PcsB, and D-alanyl-D-alanine-carboxypeptidase, putative enzymes involved in metabolism of carbohydrates including 6-phosphofructokinase PfkA, isocitrate dehydrogenase Icd, and ribulose-5-phosphate 3-epimerase Rpe, and other proteins including peptidyl-prolyl cis–trans isomerase PpiA, and chaperonin large subunit GroEL (Table 1). In addition, six phage proteins were enriched in EVs including two structural proteins (phage capsid and scaffolding protein as well as a phage head protein), two DNA synthesis and assembly proteins (DNA primase and portal protein), and two hypothetical proteins. Further analysis of proteins found in *S. thermophilus* EVs and cells indicated the presence of 36 additional phage proteins including holin and endolysin (Supplementary Table 1), which are previously reported to support EVs release following damaging of the producer cell wall [12]. These results are in line with the phage particles identified in TEM pictures (Supplementary Fig. 1). The presence of phage proteins largely overlapped with the proteomic response of *S. thermophilus* 065 exposed to mitomycin C, but phage proteins were more abundant after exposure. In total 49 phage proteins were detected after exposure of which 10 proteins were enriched in EVs including phage holin (Supplementary Table 1). Further analysis of other proteins revealed that the protein composition of EVs of non-exposed and exposed *S. thermophilus* to mitomycin C were very different.

Combining all this information suggests that the EV production in this strain was associated with mild prophage activity, which formed bacteriophage particles and expressed (low levels of) cell envelop-degrading enzymes. The role of prophage activation was confirmed in an experiment with the addition of mitomycin C that induced strong expression of phage proteins including holin and endolysin and resulted in a drop in culture OD with concomitant EV and phage release (Supplementary Table 1, Supplementary Fig. 2). These results are in line with the previously reported role of holin and endolysin phage proteins in release of *L. lactis* EVs following damaging of the producer cell wall [12].

### Immune Response Assays

**Fig. 5:**
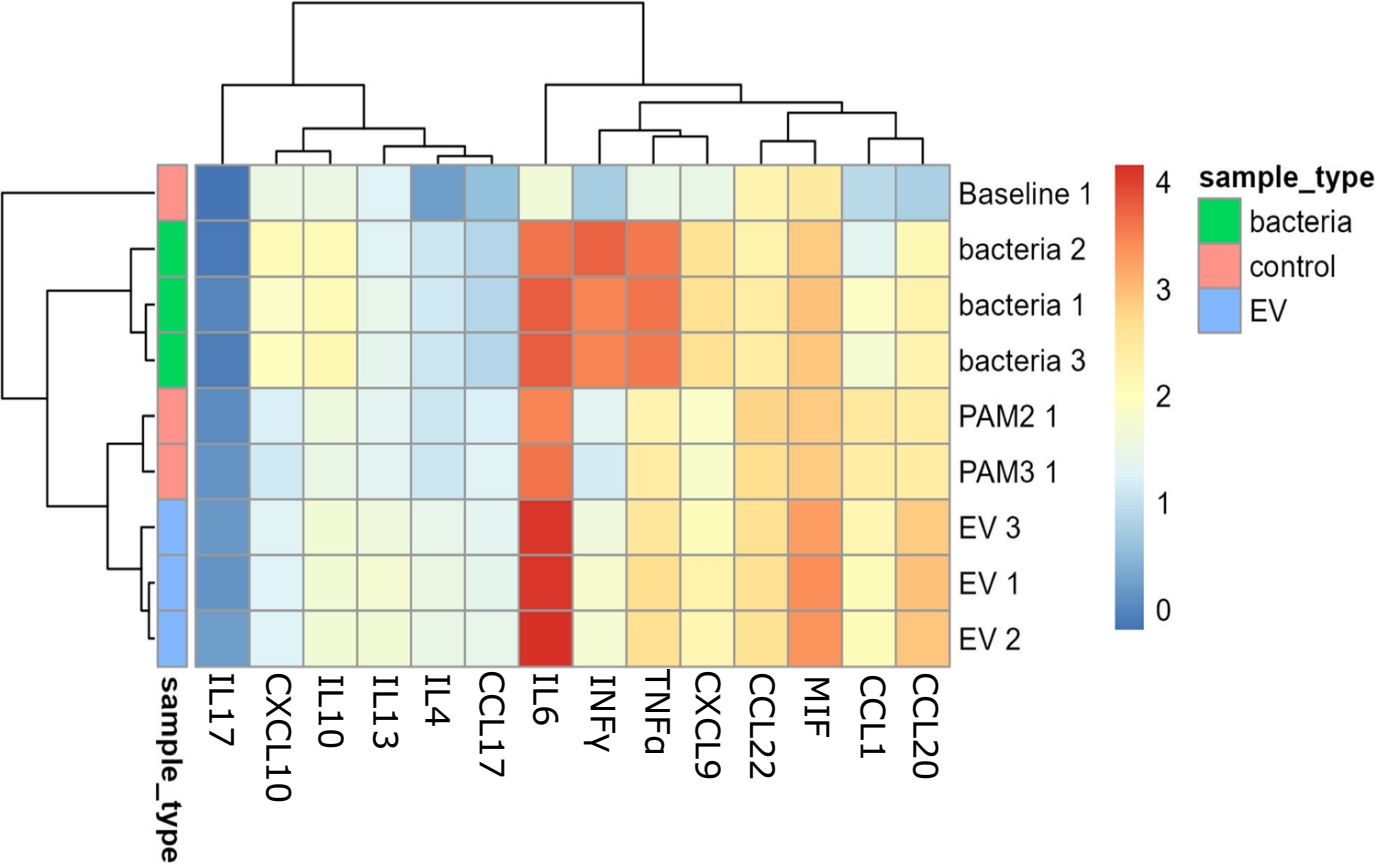
Concentration of the different cytokines and chemokines produced by non-stimulated PBMCs after exposure with *S. thermophilus* 065 bacterial cells and corresponding EVs in comparison to the positive controls (PAM 2 and PAM 3) and the baseline (medium control). The colour scale represents the log10 transformed concentration (pg/mL). The assay was performed in biological triplicates for both bacteria and EVs

To investigate if the EVs of *S. thermophilus* 065 can elicit an immune response, we exposed human peripheral blood mononuclear cells (PBMCs) to *S. thermophilus* 065 bacterial cells or the corresponding EVs. Both EVs as well as cells induced soluble mediator release (Fig. 5). However, the production of many cytokines and chemokines was significantly different between bacterial cells and EVs (*p* < 0.05, *t*-test with Bonferroni correction) (Supplementary Table 2). PBMCs exposed to EVs released significantly more CCL-20, MIF, IL-4, and CCL1, while exposure to bacterial cells resulted in significantly increased levels of IFNγ, TNFα, CXCL-10, CXCL-9, and IL-10.

**Fig. 6:**
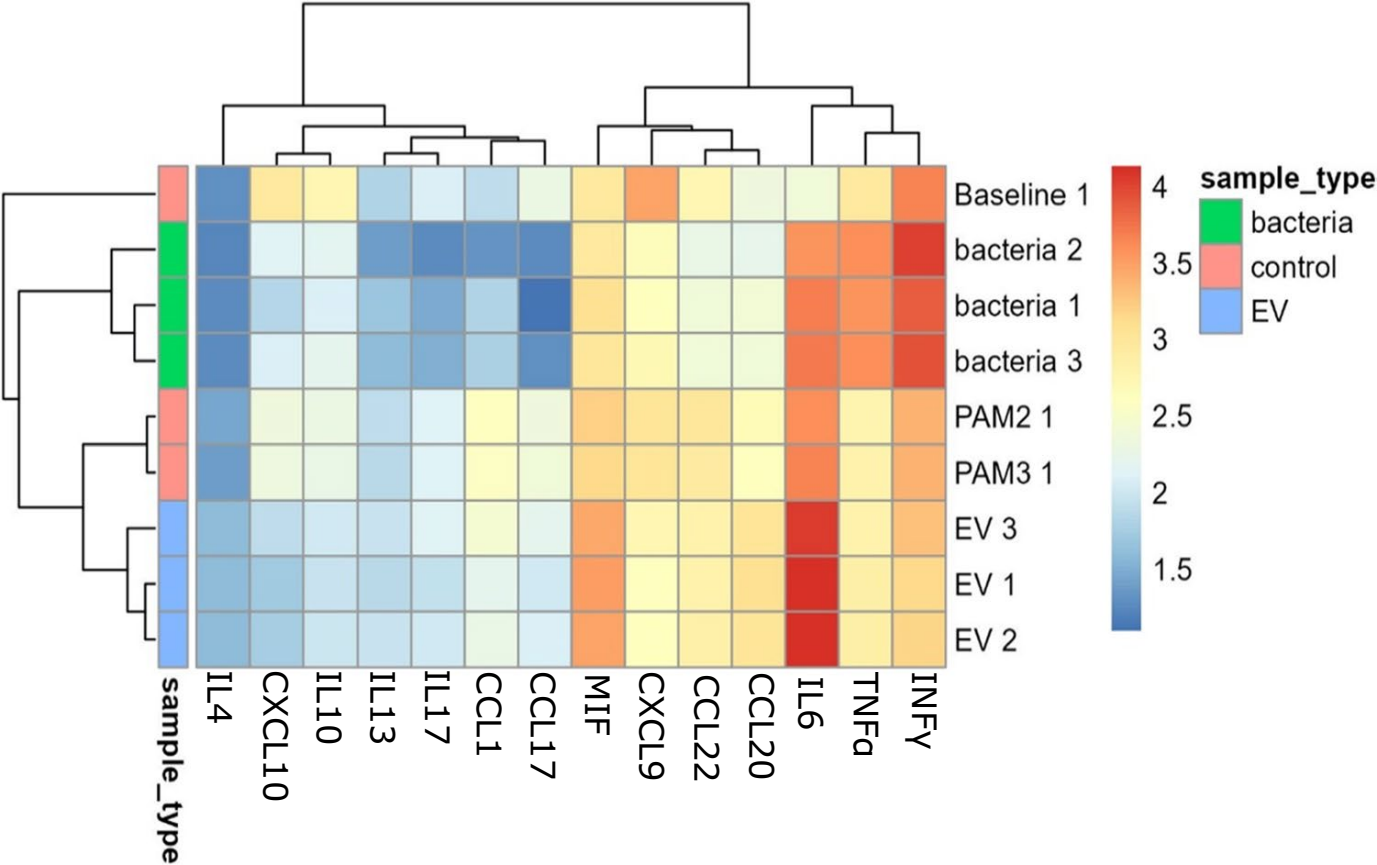
Concentration of different cytokines and chemokines produced by stimulated PBMCs after exposure with *S. thermophilus* 065 bacterial cells and corresponding EVs in comparison to the positive controls (PAM 2 and PAM 3) and the baseline (medium control). The colour scale represents the log10 transformed concentration (pg/mL). The assay was performed in biological triplicates for both bacteria and EVs

Next, we investigated whether EVs or cells could exhibit anti-inflammatory properties. To that end, PBMCs were pre-incubated with EVs or cells followed by stimulation with aCD3 and aCD28 mimicking an inflammatory response (Fig. 6). EVs and bacterial cells were both able to reduce the production of CXCL-9, IL-10 and CXCL-10 and increase the production of IL-6 (*p* > 0.05; *t*-test with Bonferroni correction) (Supplementary Table 3). In addition, bacterial cells also reduced the production of CCL-17 and CCL-22 and increased TNFα, while EVs increased the production of CCL-20, MIF and IL-4 (*p* < 0.05, *t*-test with Bonferroni correction) (Supplementary Table 3).

Proteomic analysis of EVs of mitomycin C-exposed and non-exposed *S. thermophilus* 065 revealed differences in protein profiles. However, these differences did not result in large differences in cytokine and chemokine profiles following exposure to PBMCs either under basal or stimulated conditions (Supplementary Fig. 3 and Fig. 4). A PERMANOVA analysis (Supplementary Table 4), showed that mitomycin C induction could only explain 2.4% of the profiles; also, the dosages of EVs and cells did not result in large differences in cytokine and chemokine release profiles (dosage could only explain 3.4% of the profile). In contrast the type of sample (EV or bacterial cells) and the stimulation by aCD3 and aCD28 explained 50.9 and 30.6% of the profile, respectively, indicating the robustness of the elicited response and mediator release profiles.

## Discussion

This study shows that *S. thermophilus* 065 can produce EVs following growth at 40 °C in M17 supplemented with 2% (w/v) lactose. Comparative proteome analysis revealed high abundance of specific proteins in *S. thermophilus* EVs compared to the producer cells, including membrane-associated substrate-binding proteins of ABC transporters, metabolic enzymes, ribosomal and phage proteins. This pattern of enrichment of proteins in EVs is similar to what was found in the EVs produced by *Lactiplantibacillus plantarum* [30]*.* Similarly, proteomic analysis of the EVs of *Bifidobacterium longum* showed that the most predominant groups of proteins corresponded to metabolic pathways, ribosomal proteins and ABC transporters in comparison with the proteome of the corresponding cell fraction [10]. Ribosomal proteins can also be abundant in EVs, as it was shown in Gram-negative bacteria and Gram-positive bacteria generated EVs, conceivably linked to the action of dedicated ribosomes involved in the formation of membrane proteins [31, 32].

Another group of proteins found in *S. thermophilus* 065 EVs corresponds to phage related proteins. The proteomic results of EVs showed the presence of the prophage-encoded holin-endolysin system (Supplementary Table 1). It is conceivable that formation of *S. thermophilus* 065 EVs in growing cells is due to low level prophage activation. In mitomycin C exposed cells, there was a high-level prophage activation resulting in holin-endolysin system-mediated cell lysis and explosive EV release as previously shown in *Lactococcus lactis* [12]**.** Phage holin is responsible for creating small lesions in the membrane that allows phage endolysin to access and act on the peptidoglycan leading to cell wall damage and disruption and subsequent (explosive) release of phages and EVs [33].

Previous research has shown that selected *S. thermophilus* strains undergo lysis due to prophage activation under unfavourable environmental conditions, which includes depleted lactose concentration [33–35]. It is conceivable that selection of *S. thermophilus* strains and/or fermentation conditions enable prophage induction and EV formation. However, more research needs to be done to understand mechanisms underlying the formation and functionality of EVs.

The EVs produced by beneficial bacteria are potentially important players in host-microbe interactions and could confer health benefits to the host by immune modulation [36, 37]. It has been shown that EVs produced by probiotic bacteria like *Lactiplantibacillus plantarum* [36], *Propionibacterium freudenreichii* [38], *Lactiplantibacillus reuteri* [39], and *Lactiplantibacillus lactis* [40] can also elicit a host immune response. *S. thermophilus* has been widely used for the manufacturing of dairy products but has also been recognized for its health-promoting properties which include modulation of the immune system [41, 42].

In this study, we demonstrated that *S. thermophilus* 065 EVs can elicit an immune response by immune-competent cells, as well as exhibit anti-inflammatory properties following an inflammatory trigger. Some commonalities could be observed between the soluble mediator profiles elicited by the PAM-controls and EVs which could be expected since EVs contain membrane-associated proteins and PAMs are synthetic di-, and triacylated lipopeptides that mimic the acylated amino terminus of bacterial lipoproteins [43]. Both are recognized by the pattern-recognition receptor TLR-2, which plays an important role in initiating a host-immune response [44]. Cells of *S. thermophilus* 285 have been previously shown to elicit an immune response in human monocytes by increasing the production of cytokines (IL-1α, IL-1β, IL-2, IL-6, IL-8, IL-23, IFNγ, TNFα, CSF-2) and regulating the inflammatory response in PBMCs [41, 45]. Likewise, cells of *S. thermophilus* 19 have also been shown to decrease pro-inflammatory cytokines levels in LPS-induced sepsis mouse models [46].

The *S. thermophilus* EV-modulated cytokines/chemokines are involved in different processes of signalling and cell differentiation. For instance, IL4, IL13 IL10, and IFN-γ, which are anti-inflammatory/regulatory cytokines, can modulate inflammatory processes like allergies and intestinal inflammatory diseases by regulating the immune response of the T helper 2 cells (Th2) and the T helper 1 responses (Th1) [47–49]. On the other hand, the production of pro-inflammatory cytokines can stimulate the immune system in preparation to fight pathogens and elevate the host resistance [50]. A study performed with EVs from three probiotic bacteria, *Bifidobacterium longum*, *Clostridium butyricum*, and *Lactiplantibacillus plantarum* WCFS1, revealed their capacity to elicit the production of pro-inflammatory cytokines TNFα and IL6 showing their potential capacity to act as adjuvants in immune therapy [23]. In addition, it is known that cells of *S. thermophilus* 285 can induce the production of IFNγ and TNFα, which contribute to the defence against pathogens [41]. In some cases, the production of both types of cytokines/chemokines serves to maintain immune homeostasis and to regulate the adaptive immune response of the host as seen for probiotic strains of *Escherichia coli* [51, 52].

Finally, despite the overall differences observed in the proteome cargo of the EVs obtained without and with mitomycin C added to the culture, the resulting EV-induced immune response in (non-activated and activated) PBMCs was very similar. This might indicate that the immune response in this case could be regulated in part by shared PAMs which in Gram-positive bacteria these include lipoproteins, cell wall teichoic and lipoteichoic acids, peptidoglycan fragments, certain carbohydrates and nucleic acids [53]. As mentioned above these PAMs are recognized by pattern recognition receptors (PRRs) such as TLRs inducing the immune response [54].

A minimal role in the immune response of the EVs could also be attributed to ABC-type transporters and chaperons such as GroEL, which have been reported to impact immune modulation in human cells in vitro [55–57]. Finally, phage proteins and phage particles cannot be excluded as the latter have recently been reported to interact with (human) host cells [58, 59].

## Conclusion

This study shows the production and release of EVs throughout the growth of *S. thermophilus* 065, reaching a maximum in stationary phase. Proteome analysis showed that EVs were enriched in membrane-associated substrate binding proteins, cell wall biosynthesis enzymes, metabolic enzymes, and phage related proteins. *S. thermophilus* EVs elicited an immune response in PBMCs characterized by the secretion of a range of cytokines and chemokines, and which was distinct from the range of cytokines elicited by the corresponding cells. This study indicates that EVs from *S. thermophilus* can be potentially used as postbiotics for immune modulation of the host. Obviously, additional biochemical analysis and characterization are required to elucidate and quantify the contributions of specific components of EVs and combinations thereof in in vitro studies using different cell types. Further studies are also required to elucidate the immune modulation capacity of the EVs in vivo in suitable model systems.

## Supplementary Information

Below is the link to the electronic supplementary material.

Supplementary file1 (DOCX 1.18 MB)

Supplementary file2 (XLSX 450 KB)
