# Supplementary material for: Characterization of Extracellular Vesicles from Streptococcus thermophilus 065 and Their Potential to Modulate the Immune Response
Source: Probiotics Antimicrob Proteins. 2025 Feb 1;17(4):2301–12. doi: 10.1007/s12602-024-10422-0 (PMC12405351; doi:10.1007/s12602-024-10422-0)
Supplement: Supplementary file 1 — Supplementary file1 (DOCX 1.18 MB) [file 12602_2024_10422_MOESM1_ESM.docx]

**Supplementary figures and tables**

Title: Characterization of extracellular vesicles from *Streptococcus thermophilus* 065 and their potential to modulate the immune response

Authors: Angela Rocio Ortiz Camargo^a^, Oscar van Mastrigt^a^, Joost W. Gouw^b^, Yue Liu^a^, Roger S. Bongers^b^, Jeroen van Bergenhenegouwen ^b^, Jan Knol^b,c^, Tjakko Abee^a^, Eddy J. Smid^a^

^a^ Food Microbiology, Wageningen University & Research, PO Box 17, 6700 AA Wageningen, The Netherlands.

^b^ Danone Research, Uppsalalaan 12, 3584 CT Utrecht, The Netherlands.

^c^ Laboratory of Microbiology, Wageningen University & Research, Wageningen

Corresponding author: Eddy J. Smid, PO Box 17, 6700AA Wageningen, The Netherlands. [eddy.smid@wur.nl](mailto:eddy.smid@wur.nl), +31317482834.


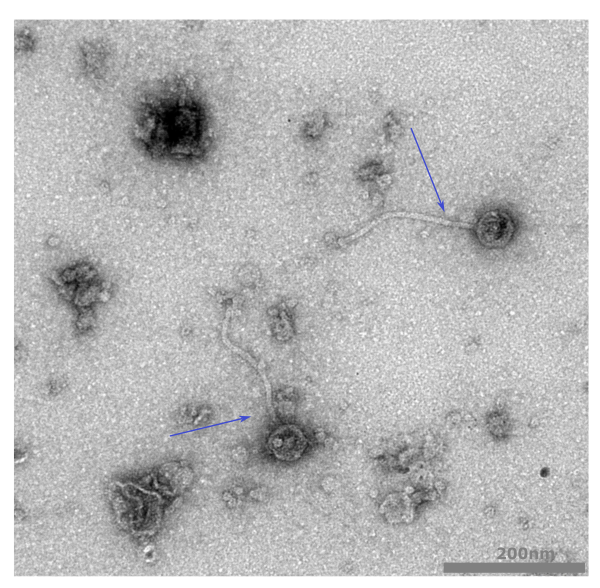


**Supplementary Fig. 1** Transmission electron microscopy of *S. thermophilus* phage particles in suspension.

A B


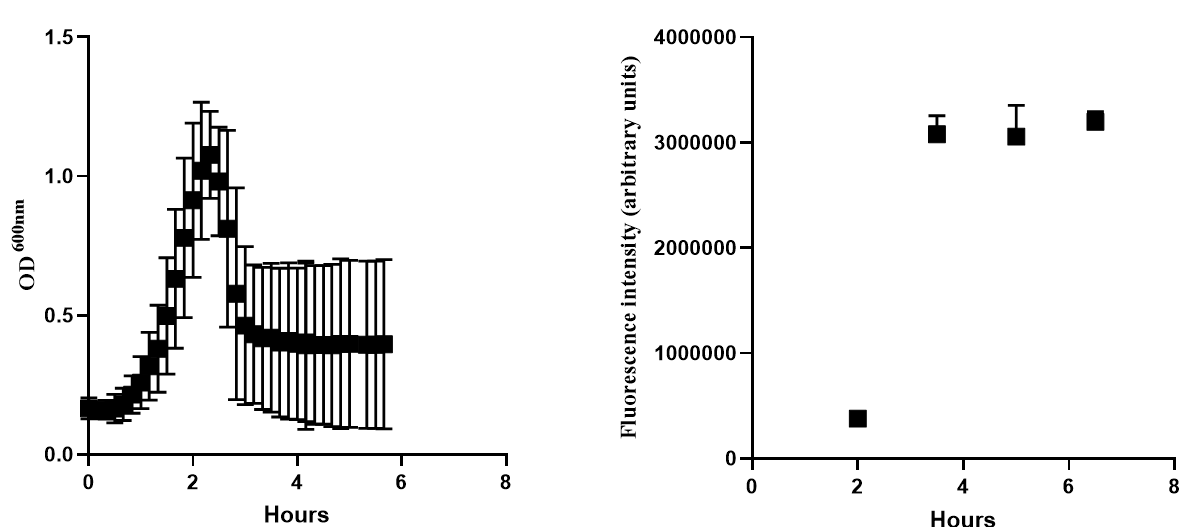


**Supplementary Fig. 2** Growth of *S. thermophilus* in presence of mitomycin C **B.** EV production of *S. thermophilus* in the presence of mitomycin C as indicated by the fluorescence of FM4-64. Error bars correspond to the standard error of the mean.


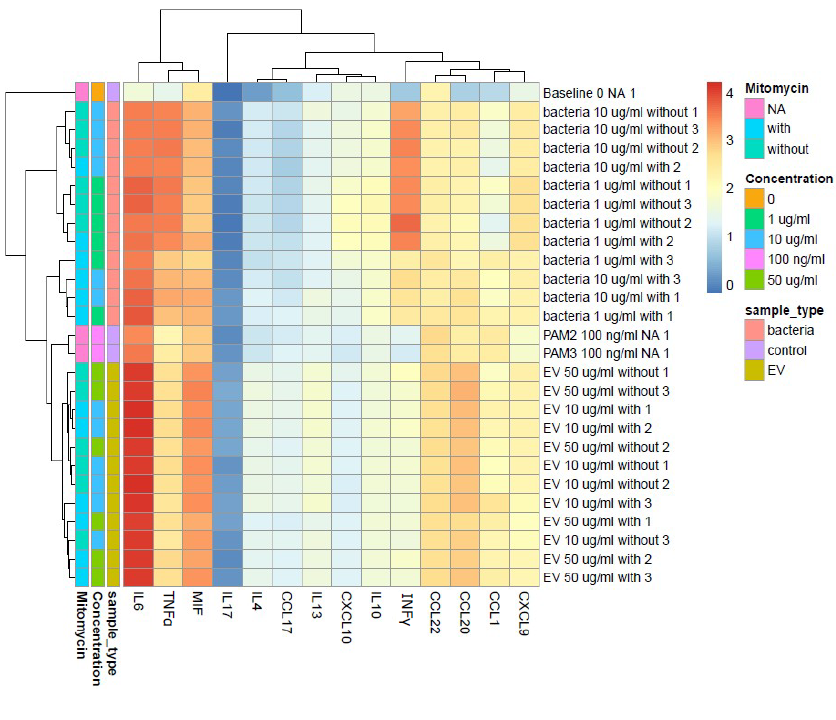


**Supplementary Fig. 3** Concentration of the different cytokines and chemokines produced by non-stimulated PBMCs after exposure with different concentrations of *S. thermophilus* bacterial cells and corresponding EVs that were non-induced or induced with mitomycin C. PAM2 and PAM3 are positive controls and the baseline is a medium control. The colour scale represents the log10 transformed concentration (pg/ml). The assay was performed in biological triplicates for both bacteria and EVs.


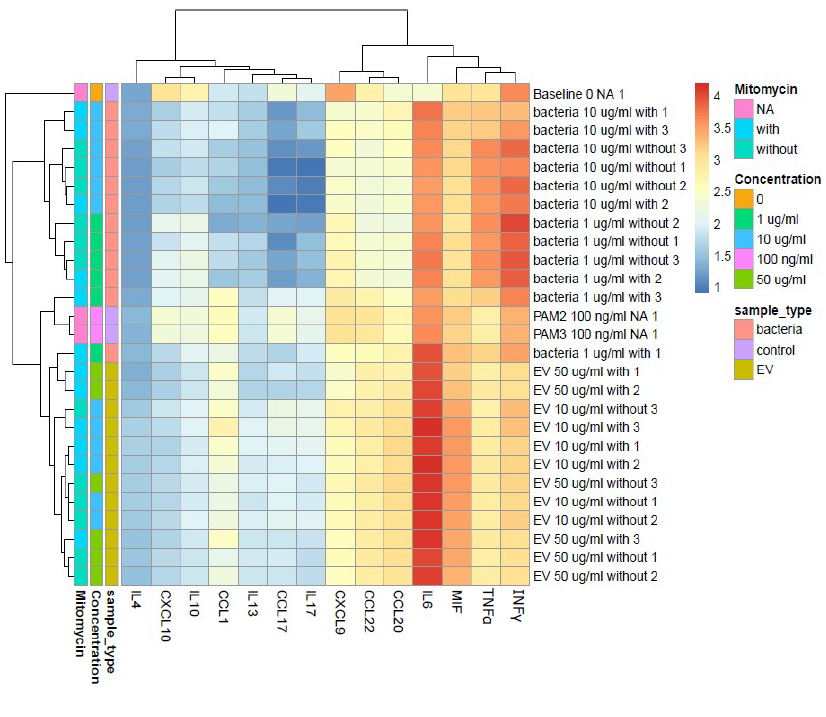


**Supplementary Fig. 4** Concentration of the different cytokines and chemokines produced by stimulated PBMCs after exposure with different concentrations of *S. thermophilus* bacterial cells and corresponding EVs that were non-induced or induced with mitomycin C. PAM2 and PAM3 are positive controls and the baseline is a medium control. The colour scale represents the log10 transformed concentration (pg/ml). The assay was performed in biological triplicates for both bacteria and EVs.

| **Marker** | **Difference mean (log10)** | **Standard error** | **Fold change** | **Adjusted p** |
| --- | --- | --- | --- | --- |
| TNFα | -0.95 | 0.06 | 0.11 | 0.003 |
| CXCL9 | -0.44 | 0.02 | 0.36 | 0.003 |
| INFγ | -1.85 | 0.10 | 0.01 | 0.003 |
| IL10 | -0.42 | 0.02 | 0.38 | 0.003 |
| IL4 | 0.36 | 0.03 | 2.30 | 0.008 |
| CCL20 | 0.61 | 0.05 | 4.07 | 0.011 |
| CXCL10 | -0.71 | 0.07 | 0.20 | 0.011 |
| MIF | 0.43 | 0.04 | 2.72 | 0.014 |
| CCL1 | 0.23 | 0.03 | 1.71 | 0.034 |
| CCL22 | 0.30 | 0.04 | 1.98 | 0.050 |
| IL6 | 0.33 | 0.06 | 2.14 | 0.154 |
| IL13 | 0.11 | 0.02 | 1.30 | 0.210 |
| IL17 | 0.40 | 0.11 | 2.51 | 0.672 |
| CCL1 | -0.43 | 0.18 | 0.37 | 1.000 |

**Supplementary Table 2** Differences in the concentrations of cytokines and chemokines produced by non-stimulated PBMCs after exposure with *S. thermophilus* bacterial cells and corresponding EVs. T-test were performed on the log10 transformed concentrations (with biological triplicates) and p-values were adjusted with Bonferroni correction (n=28; 2 conditions, 14 markers).

**Supplementary Table 3** Differences in the concentrations of cytokines and chemokines produced by stimulated PBMCs after exposure with *S. thermophilus* bacterial cells and corresponding EVs. T-test were performed on the log10 transformed concentrations (with biological triplicates) and p-values were adjusted with Bonferroni correction (n=28; 2 conditions, 14 markers). Concentrations were also compared to the baseline (medium control) to identify if the bacterial cells and EVs increased or decreased the cytokine and chemokine concentration compared to the baseline and/or if there was a shared response of bacterial cells and EVs.

| **Marker** | **Difference mean (log10)** | **Standard error** | **Fold change** | **Adjusted p** | **Compared to baseline** |
| --- | --- | --- | --- | --- | --- |
| IL6 | 0.39 | 0.06 | 2.43 | 0.073 | shared increase |
| MIF | 0.47 | 0.03 | 2.93 | 0.006 | increase EVs |
| TNF | -0.74 | 0.03 | 0.18 | 0.003 | increase bacterial cells |
| INF | -0.74 | 0.07 | 0.18 | 0.112 | - |
| CXCL9 | -0.01 | 0.05 | 0.97 | 1.000 | shared decrease |
| CCL22 | 0.48 | 0.04 | 3.05 | 0.006 | decrease bacterial cells |
| CCL20 | 0.61 | 0.06 | 4.05 | 0.017 | increase EVs |
| IL10 | -0.15 | 0.04 | 0.70 | 0.532 | shared decrease |
| CXCL10 | -0.23 | 0.11 | 0.59 | 1.000 | shared decrease |
| CCL1 | 0.66 | 0.18 | 4.61 | 0.588 | - |
| CCL17 | 0.84 | 0.06 | 6.94 | 0.006 | decrease bacterial cells |
| IL17 | 0.62 | 0.09 | 4.21 | 0.073 | - |
| IL13 | 0.28 | 0.04 | 1.90 | 0.062 | - |
| IL4 | 0.35 | 0.02 | 2.23 | 0.003 | increase EVs |

**Supplementary Table 4** Effect of the different factors on the immune response elicited by PBMCs as determined using PERMANOVA. The following factors were included in the analysis: type of sample (bacterial calls or EVs), stimulation (stimulation of PBMCs by aCD3 and aCD28 or not), mitomycin C (prophage induction by mitomycin C or not), dosage (concentration of bacterial cells and EVs).

|  | **Df** | **SS** | **R^2^** | **F** | **Pr(˃F)** |
| --- | --- | --- | --- | --- | --- |
| **Type of sample** | 1 | 0.105 | 0.509 | 166.70 | 0.001*** |
| **Stimulation** | 1 | 0.063 | 0.306 | 100.13 | 0.001*** |
| **Mitomycin C** | 1 | 0.005 | 0.024 | 7.74 | 0.003** |
| **Dosage** | 2 | 0.007 | 0.034 | 5.59 | 0.001*** |
| **Residuals** | 42 | 0.026 | 0.128 |  |  |
| **Total** | 47 | 0.207 | 1.000 |  |  |
